# Periodontal Diseases: Major Exacerbators of Pulmonary Diseases?

**DOI:** 10.1155/2021/4712406

**Published:** 2021-11-02

**Authors:** Bakey Kouanda, Zeeshan Sattar, Patrick Geraghty

**Affiliations:** ^1^Department of Medicine, State University of New York Downstate Health Sciences University, 450 Clarkson Avenue, Brooklyn, NY 11203, USA; ^2^Department of Cell Biology, State University of New York Downstate Health Sciences University, 450 Clarkson Avenue, Brooklyn, NY 11203, USA

## Abstract

Periodontal diseases are a range of polymicrobial infectious disorders, such as gingivitis and periodontitis, which affect tooth-supporting tissues and are linked to playing a role in the exacerbation of several pulmonary diseases. Pulmonary diseases, such as pneumonia, chronic obstructive pulmonary disease (COPD), asthma, tuberculosis, COVID-19, and bronchiectasis, significantly contribute to poor quality of life and mortality. The association between periodontal disease and pulmonary outcomes is an important topic and requires further attention. Numerous resident microorganisms coexist in the oral cavity and lungs. However, changes in the normal microflora due to oral disease, old age, lifestyle habits, or dental intervention may contribute to altered aspiration of oral periodontopathic bacteria into the lungs and changing inflammatory responses. Equally, periodontal diseases are associated with the longitudinal decline in spirometry lung volume. Several studies suggest a possible beneficial effect of periodontal therapy in improving lung function with a decreased frequency of exacerbations and reduced risk of adverse respiratory events and morbidity. Here, we review the current literature outlining the link between the oral cavity and pulmonary outcomes and focus on the microflora of the oral cavity, environmental and genetic factors, and preexisting conditions that can impact oral and pulmonary outcomes.

## 1. Introduction

**Figure 1:**
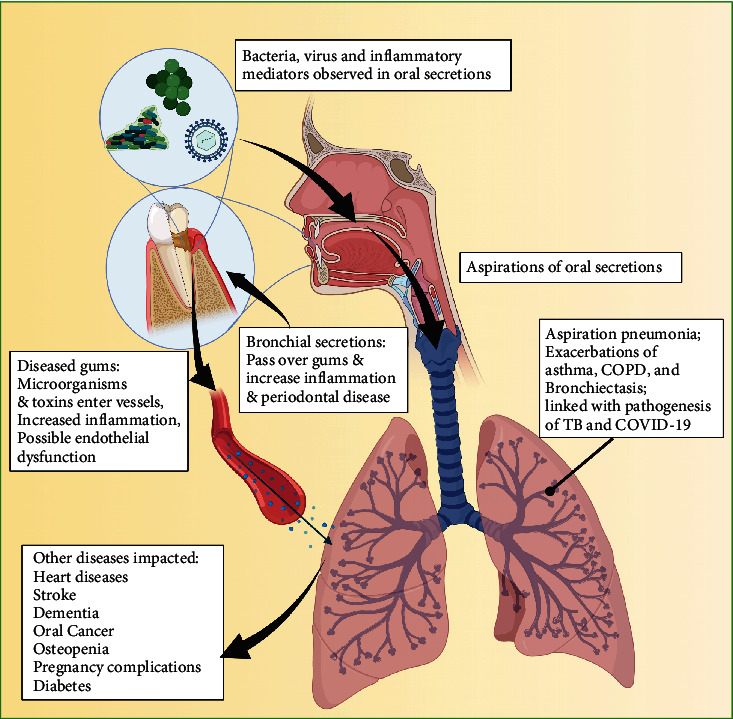
Possible interactions associating periodontal diseases with pulmonary diseases. Created with BioRender.com.

Periodontal disease, also known as gum disease, is defined as a range of polymicrobial infectious disorders (such as gingivitis and periodontitis) that affect the tooth-supporting tissues [1]. Commonly caused by bacteria and altered inflammation in the gums, gingivitis can lead to the development of periodontitis that results in a buildup of plaque on the teeth that advances towards the gum and leads to tooth loss and bone damage [2]. While being mostly preventable through good oral hygiene through brushing, flossing the teeth, and frequent visits to a dental care provider, most adults suffer from some form of gingivitis or more severe periodontitis [3]. Periodontal disease is the most common cause of tooth loss [3] and contributes to systemic diseases [4], such as pulmonary disease exacerbation [5]. Studies focused on the association of oral health diseases and lung pathology are more frequently reported over the last few years, such as pneumonia, chronic obstructive pulmonary disease (COPD), asthma, tuberculosis, COVID-19, and bronchiectasis. Importantly, periodontal disease is also associated with the longitudinal decline in spirometry lung volume [6–8]. From 2005 to 2015, over 2.7 million deaths were contributed to lower respiratory infections [9] and pulmonary diseases are a major contributor to global mortality and financial burden. Therefore, infections originating from periodontal disease could contribute to loss of lung function and possibly mortality. Here, we will review the current literature outlining the link between the oral cavity and pulmonary outcomes and focus on the microflora of the oral cavity, environmental and genetic factors, and preexisting conditions that can impact oral and pulmonary outcomes (summarized in Figure 1).

## 2. Oral and Dental Plaque Microbiome Composition Changes during Periodontitis

Microorganisms dwell in every part of our body space and are represented as symbiotic, commensal, and pathogenic microorganisms [10]. A disruption in the natural flora of the microbiome, the community of microbial residents in our body, could impact disease initiation and progression. The oral cavity houses the second largest and diverse microbial community in the body [11]. The oral cavity has many surfaces that microorganisms colonize: the teeth, tongue, cheeks, gingival sulcus, tonsils, hard palate, and soft palate [12]. The temperature (37°C), moisture, and pH (6.5-7) of the oral cavity provide an ideal environment for microbial growth [13]. The normal oral cavity microbiome contains mostly bacteria, but also fungi, viruses, archaea, and protozoa [12]. Over 700 species of prokaryotes are identified in the oral cavity, including *Firmicutes*, *Fusobacteria*, *Proteobacteria*, *Actinobacteria*, *Bacteroidetes*, *Chlamydiae*, *Chloroflexi*, *Spirochaetes*, *Synergistetes*, *Saccharibacteria*, and *Gracilibacteria* [14]. The diversity of the flora depends on the site of the oral cavity, with the tongue containing numerous papillae with few anaerobic sites and harboring mostly anaerobes [15]. Many fungal species are also present within the oral environment, such as *Candida*, *Cladosporium*, *Aureobasidium*, *Saccharomycetales*, *Aspergillus*, *Fusarium*, and *Cryptococcus* [16]. Oral health is heavily influenced by the microbiota, with the flora aiding in maintaining oral homeostasis [17]. This includes roles in food digestion, energy generation, maturation of host mucosa, immune responses, fat storage and metabolism, detoxification, providing a barrier (biofilm), and promoting and preventing microorganism colonization [10]. The periodontal microbiota is particularly heterogeneous and houses over 400 species [18]. Dysbiosis or loss of beneficial and commensal microbes that prevent the colonization of opportunistic pathogens is associated with local inflammation and exacerbation of periodontal diseases [19].

It is believed that periodontal pathogenesis depends on tooth-borne microbial biofilms/dental plaque resulting in altered host inflammation and subsequent periodontal destruction and tooth loss [20]. Therefore, many studies investigate the composition of dental plaque to determine microorganism profile changes in healthy versus diseased subjects [21, 22]. In a recent metagenomic study, the DNA from saliva and subgingival samples of 21 healthy and 48 diseased subjects identified 28 out of 1979 operational taxonomic units (OTUs) that were overabundant in diseased plaques and 6 in saliva [11]. OTU is a measurement to classify bacteria based on the sequence similarity of the 16S marker gene. Twelve OTUs were abundant in healthy plaques [11]. *Bacteroidetes*, *Spirochaetes*, *Actinobacteria*, *Veillonella*, *Capnocytophaga*, and, *Leptotrichia* were higher in healthy plaque, while *Porphyromonas*, *Tannerella*, *Prevotella*, *Filifactor*, *Mycoplasma*, *Phocaeicola*, *Johnsonella*, *Desulfobulbus*, and *Mogibacterium* were elevated in diseased plaque [11]. This study [11] and others suggest [21, 22] that there are distinctions between taxa and assembly processes of the oral microbiota between periodontal health and disease. Importantly, nonsurgical periodontal treatment restored most of the flora to healthy levels [11]. Therefore, restoration of microbiome homeostasis may be a critical step in periodontal health.

Colonization of the tooth surface leading to the formation of dental plaques begins with highly specific interactions between oral bacteria, such as *Streptococcus* species, and the tooth pellicle [23]. *Streptococcus mutans* is viewed as a primary causative agent of dental plaque formation on tooth surfaces as it is acid-tolerant [24]. It synthesizes adhesive glucan from sucrose via glucosyltransferases, and the glucan mediates adherence of the bacterium to the surface of teeth [25]. Multiple glucan-binding proteins [26] and protein antigen C [27] also participate in bacterial adherence to tooth surfaces. Together, these bacterial surface proteins coordinate to produce dental plaques, thus inducing dental caries. Similarly, *Porphyromonas gingivalis* is known to be a major player in the initiation and progression of periodontal disease [28]. More in-depth discussion of these topics is found in these review papers [29–32].

## 3. Environmental and Predisposing Factors That Alter Periodontal Health

Genetic and environmental factors can influence the oral microbiome [33] and directly contribute to periodontal pathogenesis. Here, we will briefly discuss the major factors associated with periodontal health and disease outside of day-to-day oral care.

### 3.1. Cigarette Smoke

Cigarette smoke inhalation is one of the leading risk factors for global disease burden, with smoking a major contributor to the development and progression of cancer, cardiovascular disease, chronic obstructive pulmonary disease, and periodontal disease [34]. Epidemiological studies demonstrate a significantly higher risk for periodontal disease in smokers compared to nonsmokers, which is proportional to smoking history [35–37]. Smokers exhibit greater levels of bone destruction, tooth loss [38–40], and worse clinical treatment outcomes of nonsurgical and surgical therapies [41, 42]. Smoking increases the host's susceptibility and risk of infection by inducing immune dysfunction [43, 44]. Cigarette smoke and its components do not impact microbial growth [45]. However, smoking can change virulence factors of bacteria such as altering major fimbrial antigen responses of *P. gingivalis* to TLR2 responses [45], smoking decreases IL-6 and TNF-*α* responses to *P. gingivalis* biofilms [46], and nicotine enhances *S. mutans* biofilm formation and biofilm metabolic activity [47]. Cigarette smoke and its components can trigger responses, such as matrix metalloproteinases (MMPs) and tissue inhibitors of metalloproteinases (TIMPs), to enhance collagen degradation and bone resorption [48–50]. Periodontally healthy smokers have a highly diverse subgingival biofilm, with the colonization of periodontal pathogens belonging to the genera *Fusobacterium*, *Cardiobacterium*, *Synergistes*, and *Selenomonas* [51]. When analyzing subgingival plaque samples from 200 systemically and periodontally healthy smokers and nonsmokers by 16S pyrotag sequencing, two distinct microbial composition clusters appear that separate smokers from nonsmokers [52]. In general, smokers appear to have a higher abundance of pathogenic species [53, 54]. OTUs belonging to the genera *Fusobacterium*, *Prevotella*, and *Selenomonas* were more abundant in smokers, while *Peptococcus* and *Capnocytophaga* were more abundant in nonsmokers [55, 56]. Smoking cessation reduces the risk of the onset and progression of periodontal disease [57]. Finally, smoking can impair the chemotaxis and phagocytosis of neutrophils in the periodontium [58]. This is important as approximately 30,000 polymorphonuclear neutrophils transit through periodontal tissue every minute [59] and is critical for bacterial clearance. Therefore, the immune system is constantly interacting with plaques, and factors like cigarette smoke directly impact immune responses.

### 3.2. Diabetes and Weight Gain

The risk of periodontitis is increased by approximately threefold in type 2 diabetes mellitus subjects compared with nondiabetic individuals [60, 61]. Type 1 diabetes mellitus also has an increased risk of periodontitis, with one early study in the 80s identifying that around 10% of children (<18 years) with type 1 diabetes mellitus had increased attachment loss and bone loss but with similar plaque scores [62]. This was subsequently confirmed in a larger cohort of 350 diabetic children (6-18 years old) vs. 350 nondiabetic controls, again with periodontitis observed at a greater frequency in children with diabetes (>20% vs. 8%, respectively) [63]. Other lifestyle factors such as obesity, physical activity, and diet are all linked to the risk of periodontitis, as obese rats with periodontitis had more alveolar bone loss compared with nonobese rats [64] and physical activity in humans is associated with periodontitis [65]. Equally, individuals with a BMI of ≥30 kg/m^2^ have a significantly higher risk of periodontitis compared with having a BMI of 18.5–24.9 kg/m^2^ [66]. This is also associated with insulin resistance [67]. Periodontitis can also influence glycemic control [68] and hemoglobin A1c (HbA1c) levels [69]. Equally, periodontal therapy can result in reduced HbA1c [70, 71]. Inflammation is a central feature of the pathogenesis of both diabetes and periodontitis and could impact microbial clearance and microbiome composition in the dental plaque. Type 2 diabetics have elevated levels of *P. gingivalis* [72] and type 1 diabetics have elevated *P. gingivalis* and *P. intermedia* in their oral cavity compared to nondiabetics [73].

### 3.3. Other Confounding Factors

There are several other confounding factors associated with periodontitis that we will very briefly outline here, including age, stress, medication, tooth grinding, cardiovascular disease, rheumatoid arthritis, and poor nutrition/diet. Several of these factors can be influenced by social inequalities [74]. The severity of periodontitis also increases with advancing age [75]. In an epidemiological study, the highest prevalence of chronic periodontitis was found in the elderly population (82%), followed by adults (73%) and adolescents (59%) [76].

## 4. Is There Direct Evidence Linking Periodontitis to the Exacerbation of Pulmonary Diseases?

### 4.1. Pneumonia

Hospital-acquired pneumonia (HAP) and community-acquired pneumonia (CAP) are associated with high morbidity and mortality in both healthy and vulnerable individuals and significantly contribute to the healthcare economic burden. HAP is defined as pneumonia that develops 48 hours after hospital admission or within 10 days after discharge without incubation [77], while CAP refers to an acute infection of the lungs in individuals who were not recently hospitalized and are frequently exposed to the healthcare system [78]. *Streptococcus pneumoniae* and *Haemophilus influenzae* are two of the major causes of pneumonia. However, pneumonia is also caused by several viral and bacterial pathogens observed in periodontitis, such as *Mycoplasma pneumoniae*, *Chlamydia pneumoniae*, *Legionella pneumophila*, *Porphyromonas gingivalis*, and *Treponema denticola* [79]. Plaque biofilms, the presence of certain pathogens, and periodontal-associated inflammation may contribute to the development and progression of pneumonia [80]. There is a correlation between dentition status and aspiration pneumonia in the elderly population [81]. However, a recent prospective Korean study involving datasets of 363,541 participants suggested that chronic periodontitis may not be a potential risk factor for CAP [82]. Alternatively, de Melo Neto et al. demonstrated that moderate and severe periodontitis are associated with CAP [83]. Many studies also investigated the impact of periodontal disease on various other types of pneumonia such as aspiration pneumonia and ventilator-associated pneumonia [84–86]. There is evidence to suggest that factors associated with periodontitis can influence pneumonia, but further studies are required to determine the direct impact of periodontitis on each type of pneumonia.

### 4.2. Chronic Obstructive Pulmonary Disease (COPD)

COPD is the fourth leading cause of death in the US. The CDC attributes over 480,000 deaths annually or about 1 in 5 deaths to cigarette smoke [87]. Exposure to cigarette smoke is the primary environmental factor associated with the development of COPD in the developed world. In America, it is estimated that over 30 million people have COPD, and COPD progression is linked to periodontitis. Periodontal bacteria *A. actinomycetemcomitans*, *Actinomyces*, *P gingivalis*, and *Pseudomonas aeruginosa* are detected in COPD patients [88, 89]. An investigation into the saliva microbiome of patients with both COPD and periodontitis or just periodontitis alone [90] found several members of the bacterial family *Lachnospiraceae*, such as *Rothia*, *Veillonella*, and *Actinomyces*, in patients with both conditions. This suggests that the salivary microbiome of patients with periodontitis, with or without COPD, has significantly different salivary microbiomes. A meta-analysis [91] combining data from 14 different studies concluded that COPD patients suffer from significantly worse periodontal health status with deeper periodontal pockets, worse oral hygiene, more inflammation, and more bleeding in the oral tissue. A recent study, utilizing 16S rRNA gene metagenomic sequencing, demonstrated that COPD patients without periodontitis have a decrease in bacteria richness and diversity in the periodontal microenvironment [92]. *Johnsonella*, *Campylobacter*, and *Oribacterium* were associated with COPD without periodontitis, while *Dysgonomonas*, *Desulfobulbus*, *Catonella*, *Porphyromonas endodontalis*, *Dysgonomonas wimpennyi*, *Catonella morbi*, and *Prevotella intermedia* were enriched in COPD with periodontitis [92].

### 4.3. Asthma

Asthma is a disease that manifests as chronic inflammation of the airways, with recurrent and reversible episodes of dyspnea, chest stiffness, coughing, and wheezing [93]. With asthma, the respiratory airways become inflamed and mucus production is increased and it is commonly triggered by allergies that can be relieved with treatment. Asthma affects over 235 million people per year [94]. A positive association between periodontitis and asthma was found in a Korean study consisting of 5976 patients aged 19 years and older (adjusted odds ratio: 5.36) [95]. They also observed that 83% of the patients on regular asthmatic medications were less likely to have a diagnosis of periodontitis [95]. Equally, in a Brazilian study, researchers observed a strong association with periodontitis occurring frequently in asthmatics, with an adjusted odds ratio of 4.88 [96]. They also observed that periodontitis patients were five times more likely to have bronchial inflammation than those without periodontal tissue infection [96]. Surprisingly, in an animal model of both periodontitis and asthma, researchers reported that periodontitis in asthmatic mice resulted in reduced migration of eosinophils, lymphocytes, and macrophages into the airways, reduced levels of IL-4 and TNF-*α*, and decreased mucus production [97]. The underlying mechanisms of these diseases are unclear, and further studies are needed.

### 4.4. Tuberculosis

Tuberculosis (Tb), a chronic granulomatous disease that affects the lungs, skin, bone, lymph nodes, kidneys, and oral cavity, is also linked to periodontal diseases [98]. Tb is a major cause of global death, and chemotherapeutic agents used to treat Tb can result in tuberculous lesions in the oral cavity [99]. Therefore, Tb is a predisposing/contributing factor in the pathogenesis of periodontal disease. *Mycobacterium Tb* is detected in the plaque and saliva of Tb patients with periodontitis [100, 101]. A recent study in India determined that Tb patients at 51-60 years old show a high prevalence of periodontitis, especially in males [102]. However, no study has investigated the impact of periodontal diseases on pulmonary outcomes of Tb.

### 4.5. Bronchiectasis

Patients with bronchiectasis, a chronic irreversible lung disease with permanent bronchi dilation that causes high morbidity and reduced quality of life, suffer from recurrent acute exacerbations with cough and sputum production [103]. Little direct data suggests that periodontal diseases impact bronchiectasis. However, there is an interventional randomized clinical trial underway (ClinicalTrials.gov Identifier: NCT02514226) looking at the impact of periodontitis treatment on exacerbations of bronchiectasis [104]. The same study groups amended the trial with the microbiological evaluation listed as the primary outcome (ClinicalTrials.gov Identifier: NCT02514226) [103]. However, their findings were not published at the time of this review submission. A recent study identified different inflammation profiles when comparing gingival tissues of bronchiectasis patients having chronic periodontitis, with 7 genes significantly altered in bronchiectasis patients (*LTA*, *LTB*, *TNFSF4*, *TNFSF11*, *TNFSF13*, *TNFSF13B*, and *TNFRSF11B*) [105]. Therefore, bronchiectasis could also influence oral cavity inflammation.

### 4.6. COVID-19

Oral cavities have come under renewed scrutiny as saliva contains a significant infective SARS-CoV-2 load [106] and angiotensin-converting enzyme (ACE) 2, a host receptor for SARS-CoV-2, and the proteases responsible for viral entry are well expressed on the tongue and other oral mucosae [107]. Therefore, the oral cavity is an important site of SARS-CoV-2 replication and propagation. This is further evident with a large number of COVID-19 patients experiencing taste and smell disorders [106]. There are several other oral clinical manifestations during COVID-19, including ulcers, vesicles, vesicular bleeding, and oral candidiasis [108]. *Pseudomonas aeruginosa* and *Klebsiella pneumoniae* are detected in BALF and sputum of COVID-19-positive patients [109]. Equally, patients with severe COVID-19 undergoing invasive ventilation for ARDS have infections with *Staphylococcus aureus*, *Haemophilus influenzae*, and *Streptococcus pneumoniae* [110]. Bacteria associated with the oral cavity, such as *Veillonella*, *Prevotella*, *Campylobacter*, *Treponema*, and *Fusobacterium*, are also observed in the BALF of COVID-19 patients [111, 112]. It is also possible that the elderly population is more susceptible to pulmonary complications of COVID-19 due to higher aspiration as the elderly have reduced swallowing and cough reflex function. A recent retrospective case-control study demonstrates that chronic periodontitis complicates mild cases of COVID-19 [113]. Periodontitis was associated with a higher risk of ICU admission, a greater need for ventilation, and mortality of COVID-19 patients [113]. *Fusobacterium nucleatum*, an oral bacterium, induces the expression of ACE2 in alveolar epithelial cells, thereby making the lung cells more susceptible to SARS-CoV-2 infection [114]. Therefore, the oral cavity and periodontal diseases may worsen pulmonary outcomes in COVID-19 patients.

## 5. Possible Mechanisms Apart from the Presence of Bacteria?

There is some indirect mechanistic data to link periodontal disease to pulmonary disease. Periodontal disease is associated with low-grade systemic inflammation, which could affect lung function. Adverse effects of oral inflammation on lung function are suggested to start during adolescence [115]. The aspiration of bacteria from the oral cavity might be considered an underlying mechanism for oral inflammatory and lung function [115]. Periodontal pockets, which are spaces or openings surrounding the teeth under the gum line, present in disease, can promote the accumulation of dental plaques, resulting in the growth and reproduction of pathogenic bacteria [116]. Aspiration of oral pathogens into the lower airways could trigger the development of pneumonia or induce an exacerbation of asthma, COPD, or bronchiectasis. Subsequent excessive cytokines and chemokine production could further exacerbate pulmonary responses including mucus production [117]. One study [118] suggests that it is due to the physical dissemination of periodontal bacteria, with inflammation and bleeding of the gums leading to more opportunity of the bacteria to enter the bloodstream. It is through the distribution of periodontal bacteria and likely other factors in the oral cavity in the body that amplifies the overall systemic inflammatory response.

## 6. Dental Treatment and Pulmonary Outcomes

Dental treatments can have varied effects on pulmonary physiology and the course of pulmonary diseases as outlined in this section and Table 1.

### 6.1. Asthma

Routine dental treatment is linked to decreased lung function in children who have underlying asthma [119]. Colophony, which is used in some fluoride varnishes, is known to cause sensitivity reactions and thus is relatively contraindicated in children with severe asthma [120]. Similarly, dental providers should be careful while giving local anesthetics to patients due to the presence of sodium metabisulfite, as it may trigger hypersensitivity reactions that can result in asthma exacerbations [121]. Sulfite sensitivity, however, is not common and patients who exhibit such sensitivity usually have severe steroid-dependent asthma. Other triggers of asthma exacerbations arising in the setting of dental procedures include aerosols from ultrasonic handpieces, tooth enamel dust, dental material residues, and supine positioning for an extended time [119, 122, 123].

### 6.2. COPD

There is some evidence suggesting that periodontal debridement, either through ultrasonic instrumentation or hand instrumentation, does not increase the incidence of COPD or affect the quality of life of patients with preexisting COPD [124]. Other studies suggest that periodontal therapy in COPD patients with chronic periodontitis may improve lung function and decrease the frequency of COPD exacerbations [125, 126].

### 6.3. Transmission of Infections

Dental procedures and equipment can be a major source for the transmission of pathogens. *Pseudomonas aeruginosa*, a leading causative agent of nosocomial pneumonia, was detected in the water line supplying dental units like dental triple function syringes, contra-angle handpieces, and ultrasonic scalers [127]. On the other hand, some studies have shown that dental interventions, including oral hygiene by mechanical and topical chemical means, can reduce the incidence of nosocomial pulmonary infections by approximately 40% [128].

### 6.4. Aspiration Risk of Foreign Bodies

Dental manipulation entails the risk of accidental aspiration of foreign bodies. These objects can be aspirated into the trachea and cause pulmonary complications. Objects causing complete or partial obstruction of the central airways can lead to respiratory distress or arrest. Aspiration of sharp objects may lead to pneumothorax [129] or hemorrhage. Long-term complications from these instances include atelectasis [130], hyperinflation [129], recurrence of pulmonary infections, and bronchiectasis [131].

## 7. Conclusions

Alterations in the flora in the oral cavity are frequently observed in periodontal diseases and may contribute to pulmonary outcomes in several diseases. In general, oral interventional approaches appear to yield improved pulmonary outcomes, but the risk of aspiration, especially in the older population, may influence exacerbations in patients with pulmonary comorbidities. While we do not discuss in great detail here, the treatment for the lung disease could also affect oral cavity outcomes. Overall, further studies are required to determine the impact of periodontal diseases on pulmonary outcomes.

## Figures and Tables

**Table 1:** Summary of dental treatment and pulmonary outcomes.

Pulmonary diseases	Effects of dental treatment
Asthma	(i) Hypersensitivity reactions to colophony [120] and sodium metabisulfite [121] (ii) Asthma exacerbations due to aerosols from ultrasonic handpieces, tooth enamel dust, dental material residues, and supine positioning for an extended time [119, 122, 123]
COPD	(i) Decrease in COPD exacerbations [125, 126]
Infections	(i) Transmission of *Pseudomonas* infections [127] (ii) Reduction in nosocomial infections [128]
Aspiration-linked pulmonary outcomes	(i) Pneumothorax [129] (ii) Atelectasis [130] (iii) Hyperinflation [129] (iv) Bronchiectasis [131]

## References

[1] Touger-Decker R., Mobley C. C., American Dietetic Association (2003). Position of the American Dietetic Association: oral health and nutrition. *Journal of the American Dietetic Association*.

[2] Bailey D. L., Barrow S. Y., Cvetkovic B. (2016). Periodontal diagnosis in private dental practice: a case-based survey. *Australian Dental Journal*.

[3] Eke P. I., Borgnakke W. S., Genco R. J. (2020). Recent epidemiologic trends in periodontitis in the USA. *Periodontology 2000*.

[4] Buhlin K., Gustafsson A., Håkansson J., Klinge B. (2002). Oral health and cardiovascular disease in Sweden. *Journal of Clinical Periodontology*.

[5] Griffin S. O., Barker L. K., Griffin P. M., Cleveland J. L., Kohn W. (2009). Oral health needs among adults in the United States with chronic diseases. *Journal of the American Dental Association (1939)*.

[6] Hayes C., Sparrow D., Cohen M., Vokonas P. S., Garcia R. I. (1998). The association between alveolar bone loss and pulmonary function: the VA Dental Longitudinal Study. *Annals of Periodontology*.

[7] Garcia R. I., Nunn M. E., Vokonas P. S. (2001). Epidemiologic associations between periodontal disease and chronic obstructive pulmonary disease. *Annals of Periodontology*.

[8] Hamalainen P., Suominen H., Keskinen M., Meurman J. H. (2004). Oral health and reduction in respiratory capacity in a cohort of community-dwelling elderly people: a population-based 5-year follow-up study. *Gerodontology*.

[9] Ng M., Fleming T., Robinson M. (2014). Global, regional, and national prevalence of overweight and obesity in children and adults during 1980-2013: a systematic analysis for the Global Burden of Disease Study 2013. *The Lancet*.

[10] Kilian M., Chapple I. L., Hannig M. (2016). The oral microbiome - an update for oral healthcare professionals. *British Dental Journal*.

[11] Chen C., Hemme C., Beleno J. (2018). Oral microbiota of periodontal health and disease and their changes after nonsurgical periodontal therapy. *The ISME Journal*.

[12] Zaura E., Nicu E. A., Krom B. P., Keijser B. J. F. (2014). Acquiring and maintaining a normal oral microbiome: current perspective. *Frontiers in Cellular and Infection Microbiology*.

[13] Lim Y., Totsika M., Morrison M., Punyadeera C. (2017). Oral microbiome: a new biomarker reservoir for oral and oropharyngeal cancers. *Theranostics*.

[14] Perera M., al-hebshi N. N., Speicher D. J., Perera I., Johnson N. W. (2016). Emerging role of bacteria in oral carcinogenesis: a review with special reference to perio-pathogenic bacteria. *Journal of Oral Microbiology*.

[15] Sultan A. S., Kong E. F., Rizk A. M., Jabra-Rizk M. A. (2018). The oral microbiome: a lesson in coexistence. *PLoS Pathogens*.

[16] Sharma N., Bhatia S., Singh Sodhi A., Batra N. (2018). Oral microbiome and health. *AIMS Microbiology*.

[17] Mark Welch J. L., Rossetti B. J., Rieken C. W., Dewhirst F. E., Borisy G. G. (2016). Biogeography of a human oral microbiome at the micron scale. *Proceedings of the National Academy of Sciences of the United States of America*.

[18] Dewhirst F. E., Chen T., Izard J. (2010). The human oral microbiome. *Journal of Bacteriology*.

[19] Scannapieco F. A., Dongari-Bagtzoglou A. (2021). Dysbiosis revisited. Understanding the role of the oral microbiome in the pathogenesis of gingivitis and periodontitis: a critical assessment. *Journal of Periodontology*.

[20] Frédéric L., Michel B., Selena T. (2018). Oral microbes, biofilms and their role in periodontal and peri-implant diseases. *Materials (Basel)*.

[21] Liu B., Faller L. L., Klitgord N. (2012). Deep sequencing of the oral microbiome reveals signatures of periodontal disease. *PLoS One*.

[22] Wang J., Qi J., Zhao H. (2013). Metagenomic sequencing reveals microbiota and its functional potential associated with periodontal disease. *Scientific Reports*.

[23] Kolenbrander P. E., Palmer R. J., Periasamy S., Jakubovics N. S. (2010). Oral multispecies biofilm development and the key role of cell-cell distance. *Nature Reviews Microbiology*.

[24] Kawabata S., Hamada S. (1999). Studying biofilm formation of mutanss streptococci. *Methods in Enzymology*.

[25] Yamashita Y., Bowen W. H., Burne R. A., Kuramitsu H. K. (1993). Role of the Streptococcus mutans gtf genes in caries induction in the specific-pathogen-free rat model. *Infection and Immunity*.

[26] Banas J. A., Vickerman M. M. (2003). Glucan-binding proteins of the oral streptococci. *Critical Reviews in Oral Biology and Medicine*.

[27] Koga T., Okahashi N., Takahashi I., Kanamoto T., Asakawa H., Iwaki M. (1990). Surface hydrophobicity, adherence, and aggregation of cell surface protein antigen mutants of Streptococcus mutans serotype c. *Infection and Immunity*.

[28] Hajishengallis G., Liang S., Payne M. A. (2011). Low-abundance biofilm species orchestrates inflammatory periodontal disease through the commensal microbiota and complement. *Cell Host & Microbe*.

[29] Abranches J., Zeng L., Kajfasz J. K. (2018). Biology of oral streptococci. *Microbiology Spectrum*.

[30] Bowen W. H., Burne R. A., Wu H., Koo H. (2018). Oral biofilms: pathogens, matrix, and polymicrobial interactions in microenvironments. *Trends in Microbiology*.

[31] Baker J. L., Edlund A. (2018). Exploiting the oral microbiome to prevent tooth decay: has evolution already provided the best tools?. *Frontiers in Microbiology*.

[32] Hajishengallis G., Darveau R. P., Curtis M. A. (2012). The keystone-pathogen hypothesis. *Nature Reviews Microbiology*.

[33] Demmitt B. A., Corley R. P., Huibregtse B. M. (2017). Genetic influences on the human oral microbiome. *BMC Genomics*.

[34] G B D R F Collaborators (2018). Global, regional, and national comparative risk assessment of 84 behavioural, environmental and occupational, and metabolic risks or clusters of risks for 195 countries and territories, 1990-2017: a systematic analysis for the Global Burden of Disease Study 2017. *The Lancet*.

[35] Do L. G., Slade G. D., Roberts-Thomson K. F., Sanders A. E. (2008). Smoking-attributable periodontal disease in the Australian adult population. *Journal of Clinical Periodontology*.

[36] Bergstrom J. (2014). Smoking rate and periodontal disease prevalence: 40-year trends in Sweden 1970-2010. *Journal of Clinical Periodontology*.

[37] Eke P. I., Wei L., Thornton-Evans G. O. (2016). Risk indicators for periodontitis in US adults: NHANES 2009 to 2012. *Journal of Periodontology*.

[38] Ylostalo P., Sakki T., Laitinen J., Jarvelin M. R., Knuuttila M. (2004). The relation of tobacco smoking to tooth loss among young adults. *European Journal of Oral Sciences*.

[39] Baljoon M., Natto S., Bergstrom J. (2005). Long-term effect of smoking on vertical periodontal bone loss. *Journal of Clinical Periodontology*.

[40] Johnson G. K., Guthmiller J. M. (2007). The impact of cigarette smoking on periodontal disease and treatment. *Periodontology 2000*.

[41] Heasman L., Stacey F., Preshaw P. M., McCracken G. I., Hepburn S., Heasman P. A. (2006). The effect of smoking on periodontal treatment response: a review of clinical evidence. *Journal of Clinical Periodontology*.

[42] Patel R. A., Wilson R. F., Palmer R. M. (2012). The effect of smoking on periodontal bone regeneration: a systematic review and meta-analysis. *Journal of Periodontology*.

[43] Foronjy R. F., Dabo A. J., Taggart C. C., Weldon S., Geraghty P. (2014). Respiratory syncytial virus infections enhance cigarette smoke induced COPD in mice. *PLoS One*.

[44] Petrovic M., Kesic L., Obradovic R. (2013). Comparative analysis of smoking influence on periodontal tissue in subjects with periodontal disease. *Materia Socio Medica*.

[45] Bagaitkar J., Williams L. R., Renaud D. E. (2009). Tobacco-induced alterations to Porphyromonas gingivalis-host interactions. *Environmental Microbiology*.

[46] Bagaitkar J., Daep C. A., Patel C. K., Renaud D. E., Demuth D. R., Scott D. A. (2011). Tobacco smoke augments Porphyromonas gingivalis-Streptococcus gordonii biofilm formation. *PLoS One*.

[47] Huang R., Li M., Gregory R. L. (2012). Effect of nicotine on growth and metabolism of Streptococcus mutans. *European Journal of Oral Sciences*.

[48] Zhou J., Olson B. L., Windsor L. J. (2007). Nicotine increases the collagen-degrading ability of human gingival fibroblasts. *Journal of Periodontal Research*.

[49] Katono T., Kawato T., Tanabe N. (2009). Effects of nicotine and lipopolysaccharide on the expression of matrix metalloproteinases, plasminogen activators, and their inhibitors in human osteoblasts. *Archives of Oral Biology*.

[50] Kim Y. S., Shin S. I., Kang K. L. (2012). Nicotine and lipopolysaccharide stimulate the production of MMPs and prostaglandin E2by hypoxia-inducible factor-1*α* up-regulation in human periodontal ligament cells. *Journal of Periodontal Research*.

[51] Kumar P. S., Matthews C. R., Joshi V., de Jager M., Aspiras M. (2011). Tobacco smoking affects bacterial acquisition and colonization in oral biofilms. *Infection and Immunity*.

[52] Mason M. R., Preshaw P. M., Nagaraja H. N., Dabdoub S. M., Rahman A., Kumar P. S. (2015). The subgingival microbiome of clinically healthy current and never smokers. *The ISME Journal*.

[53] Joshi V., Matthews C., Aspiras M., de Jager M., Ward M., Kumar P. (2014). Smoking decreases structural and functional resilience in the subgingival ecosystem. *Journal of Clinical Periodontology*.

[54] Matthews C. R., Joshi V., de Jager M., Aspiras M., Kumar P. S. (2013). Host-bacterial interactions during induction and resolution of experimental gingivitis in current smokers. *Journal of Periodontology*.

[55] Moon J. H., Lee J. H., Lee J. Y. (2015). Subgingival microbiome in smokers and non-smokers in Korean chronic periodontitis patients. *Molecular Oral Microbiology*.

[56] Camelo-Castillo A. J., Mira A., Pico A. (2015). Subgingival microbiota in health compared to periodontitis and the influence of smoking. *Frontiers in Microbiology*.

[57] Dietrich T., Walter C., Oluwagbemigun K. (2015). Smoking, smoking cessation, and risk of tooth loss: the EPIC-Potsdam study. *Journal of Dental Research*.

[58] Guntsch A., Erler M., Preshaw P. M., Sigusch B. W., Klinger G., Glockmann E. (2006). Effect of smoking on crevicular polymorphonuclear neutrophil function in periodontally healthy subjects. *Journal of Periodontal Research*.

[59] Rindom Schiott C., Loe H. (1970). The origin and variation in number of leukocytes in the human saliva. *Journal of Periodontal Research*.

[60] Mealey B. L., Ocampo G. L. (2007). Diabetes mellitus and periodontal disease. *Periodontology 2000*.

[61] Nelson R. G., Shlossman M., Budding L. M. (1990). Periodontal disease and NIDDM in Pima Indians. *Diabetes Care*.

[62] Cianciola L. J., Park B. H., Bruck E., Mosovich L., Genco R. J. (1982). Prevalence of periodontal disease in insulin-dependent diabetes mellitus (juvenile diabetes). *Journal of the American Dental Association (1939)*.

[63] Lalla E., Cheng B., Lal S. (2007). Diabetes mellitus promotes periodontal destruction in children. *Journal of Clinical Periodontology*.

[64] Perlstein M. I., Bissada N. F. (1977). Influence of obesity and hypertension on the severity of periodontitis in rats. *Oral Surgery, Oral Medicine, and Oral Pathology*.

[65] al-Zahrani M. S., Borawski E. A., Bissada N. F. (2005). Increased physical activity reduces prevalence of periodontitis. *Journal of Dentistry*.

[66] al-Zahrani M. S., Bissada N. F., Borawski E. A. (2003). Obesity and periodontal disease in young, middle-aged, and older adults. *Journal of Periodontology*.

[67] Genco R. J., Grossi S. G., Ho A., Nishimura F., Murayama Y. (2005). A proposed model linking inflammation to obesity, diabetes, and periodontal infections. *Journal of Periodontology*.

[68] Taylor G. W., Burt B. A., Becker M. P. (1996). Severe periodontitis and risk for poor glycemic control in patients with non-insulin-dependent diabetes mellitus. *Journal of Periodontology*.

[69] Demmer R. T., Desvarieux M., Holtfreter B. (2010). Periodontal status and A1C change: longitudinal results from the study of health in Pomerania (SHIP). *Diabetes Care*.

[70] Janket S. J., Wightman A., Baird A. E., van Dyke T. E., Jones J. A. (2005). Does periodontal treatment improve glycemic control in diabetic patients? A meta-analysis of intervention studies. *Journal of Dental Research*.

[71] Darré L., Vergnes J. N., Gourdy P., Sixou M. (2008). Efficacy of periodontal treatment on glycaemic control in diabetic patients: a meta-analysis of interventional studies. *Diabetes & Metabolism*.

[72] Thorstensson H., Dahlen G., Hugoson A. (1995). Some suspected periodontopathogens and serum antibody response in adult long-duration insulin-dependent diabetics. *Journal of Clinical Periodontology*.

[73] Takahashi K., Nishimura F., Kurihara M. (2001). Subgingival microflora and antibody responses against periodontal bacteria of young Japanese patients with type 1 diabetes mellitus. *Journal of the International Academy of Periodontology*.

[74] Satcher D., Nottingham J. H. (2017). Revisiting oral health in America: a report of the surgeon general. *American Journal of Public Health*.

[75] Eke P. I., Thornton-Evans G. O., Wei L., Borgnakke W. S., Dye B. A., Genco R. J. (2018). Periodontitis in US adults: National Health and Nutrition Examination Survey 2009-2014. *Journal of the American Dental Association (1939)*.

[76] Tadjoedin F. M., Fitri A. H., Kuswandani S. O. (2017). The correlation between age and periodontal diseases. *Journal of International Dental and Medical Research*.

[77] Sopena N., Heras E., Casas I. (2014). Risk factors for hospital-acquired pneumonia outside the intensive care unit: a case-control study. *American Journal of Infection Control*.

[78] Musher D. M., Thorner A. R. (2014). Community-acquired pneumonia. *The New England Journal of Medicine*.

[79] Tan K. H., Seers C. A., Dashper S. G. (2014). Porphyromonas gingivalis and Treponema denticola exhibit metabolic symbioses. *PLoS Pathogens*.

[80] Raghavendran K., Mylotte J. M., Scannapieco F. A. (2007). Nursing home-associated pneumonia, hospital-acquired pneumonia and ventilator-associated pneumonia: the contribution of dental biofilms and periodontal inflammation. *Periodontology 2000*.

[81] Awano S., Ansai T., Takata Y. (2008). Oral health and mortality risk from pneumonia in the elderly. *Journal of Dental Research*.

[82] Kim S. J., Kim K., Choi S. (2019). Chronic periodontitis and community-acquired pneumonia: a population-based cohort study. *BMC Pulmonary Medicine*.

[83] de Melo Neto J. P., Melo M. S., dos Santos-Pereira S. A., Martinez E. F., Okajima L. S., Saba-Chujfi E. (2013). Periodontal infections and community-acquired pneumonia: a case-control study. *European Journal of Clinical Microbiology & Infectious Diseases*.

[84] Fourrier F., Cau-Pottier E., Boutigny H., Roussel-Delvallez M., Jourdain M., Chopin C. (2000). Effects of dental plaque antiseptic decontamination on bacterial colonization and nosocomial infections in critically ill patients. *Intensive Care Medicine*.

[85] Fourrier F., Dubois D., Pronnier P. (2005). Effect of gingival and dental plaque antiseptic decontamination on nosocomial infections acquired in the intensive care unit: a double-blind placebo-controlled multicenter study. *Critical Care Medicine*.

[86] Tada A., Miura H. (2012). Prevention of aspiration pneumonia (AP) with oral care. *Archives of Gerontology and Geriatrics*.

[87] U S D o H a H Services (2014). *The health consequences of smoking—50 years of progress: a report of the surgeon general*.

[88] Morris J. F., Sewell D. L. (1994). Necrotizing pneumonia caused by mixed infection with Actinobacillus actinomycetemcomitans and Actinomyces israelii: case report and review. *Clinical Infectious Diseases*.

[89] Tan L., Wang H., Li C., Pan Y. (2014). 16S rDNA-based metagenomic analysis of dental plaque and lung bacteria in patients with severe acute exacerbations of chronic obstructive pulmonary disease. *Journal of Periodontal Research*.

[90] Lin M., Li X., Wang J. (2020). Saliva microbiome changes in patients with periodontitis with and without chronic obstructive pulmonary disease. *Frontiers in Cellular and Infection Microbiology*.

[91] Shi Q., Zhang B., Xing H., Yang S., Xu J., Liu H. (2018). Patients with chronic obstructive pulmonary disease suffer from worse periodontal health-evidence from a meta-analysis. *Frontiers in Physiology*.

[92] Wu X., Chen J., Xu M. (2017). 16S rDNA analysis of periodontal plaque in chronic obstructive pulmonary disease and periodontitis patients. *Journal of Oral Microbiology*.

[93] Barnes P. J. (2008). The cytokine network in asthma and chronic obstructive pulmonary disease. *The Journal of Clinical Investigation*.

[94] Kunna R., San Sebastian M., Stewart Williams J. (2017). Measurement and decomposition of socioeconomic inequality in single and multimorbidity in older adults in China and Ghana: results from the WHO study on global AGEing and adult health (SAGE). *International Journal for Equity in Health*.

[95] Lee S. W., Lim H. J., Lee E. (2017). Association between asthma and periodontitis: results from the Korean National Health and Nutrition Examination Survey. *Journal of Periodontology*.

[96] Gomes-Filho I. S., Soledade-Marques K. R., Seixas da Cruz S. (2014). Does periodontal infection have an effect on severe asthma in adults?. *Journal of Periodontology*.

[97] Candeo L. C., Rigonato-Oliveira N. C., Brito A. A. (2017). Effects of periodontitis on the development of asthma: the role of photodynamic therapy. *PLoS One*.

[98] Sharma A., Garg H., Khattri S., Sharma S. (2016). Periodontal status of tuberculosis patients - is there a two-way link?. *The Indian Journal of Tuberculosis*.

[99] Bansal R., Jain A., Mittal S. (2015). Orofacial tuberculosis: clinical manifestations, diagnosis and management. *Journal of Family Medicine and Primary Care*.

[100] Avdonina L. I., Gedymin L. E., Erokhin V. V. (1993). Tuberculous periodontitis. *Problemy Tuberkuleza*.

[101] Palakuru S. K., Lakshman V. K., Bhat K. G. (2012). Microbiological analysis of oral samples for detection of Mycobacterium tuberculosis by nested polymerase chain reaction in tuberculosis patients with periodontitis. *Dental Research Journal*.

[102] Rastogi T., Chowdhary Z., Krishna M. K., Mehrotra S., Mohan R. (2019). Prevalence of periodontitis in patients with pulmonary disease: a cross-sectional survey in the industrial district of India. *Journal of Indian Society of Periodontology*.

[103] Pinto E. H., Longo P. L., de Camargo C. C. (2016). Assessment of the quantity of microorganisms associated with bronchiectasis in saliva, sputum and nasal lavage after periodontal treatment: a study protocol of a randomised controlled trial. *BMJ Open*.

[104] Romero S. S., Pinto E. H., Longo P. L. (2017). Effects of periodontal treatment on exacerbation frequency and lung function in patients with chronic periodontitis: study protocol of a 1-year randomized controlled trial. *BMC Pulmonary Medicine*.

[105] Gupta A., Singh N., Kumar A. (2020). Differential expression of inflammatory responsive genes between chronic periodontitis and periodontally affected bronchiectasis patients. *Molecular Biology Research Communications*.

[106] NIH COVID-19 Autopsy Consortium, HCA Oral and Craniofacial Biological Network, Huang N. (2021). SARS-CoV-2 infection of the oral cavity and saliva. *Nature Medicine*.

[107] Xu H., Zhong L., Deng J. (2020). High expression of ACE2 receptor of 2019-nCoV on the epithelial cells of oral mucosa. *International Journal of Oral Science*.

[108] Amorim dos Santos J., Normando A. G. C., Carvalho da Silva R. L. (2020). Oral mucosal lesions in a COVID-19 patient: new signs or secondary manifestations?. *International Journal of Infectious Diseases*.

[109] Huang C., Wang Y., Li X. (2020). Clinical features of patients infected with 2019 novel coronavirus in Wuhan, China. *Lancet*.

[110] Kreitmann L., Monard C., Dauwalder O., Simon M., Argaud L. (2020). Early bacterial co-infection in ARDS related to COVID-19. *Intensive Care Medicine*.

[111] Shen Z., Xiao Y., Kang L. (2020). Genomic diversity of severe acute respiratory syndrome-coronavirus 2 in patients with coronavirus disease 2019. *Clinical Infectious Diseases*.

[112] Ren L. L., Wang Y. M., Wu Z. Q. (2020). Identification of a novel coronavirus causing severe pneumonia in human: a descriptive study. *Chinese Medical Journal*.

[113] Marouf N., Cai W., Said K. N. (2021). Association between periodontitis and severity of COVID-19 infection: a case-control study. *Journal of Clinical Periodontology*.

[114] Takahashi Y., Watanabe N., Kamio N. (2021). Expression of the SARS-CoV-2 receptor ACE2 and proinflammatory cytokines induced by the periodontopathic bacterium Fusobacterium nucleatum in human respiratory epithelial cells. *International Journal of Molecular Sciences*.

[115] Heinrich J., Thiering E., Jörres R. A., Schulz H., Kühnisch J., Standl M. (2019). Lung function and oral health in adolescents. *The European Respiratory Journal*.

[116] Diaz P. I. (2012). Microbial diversity and interactions in subgingival biofilm communities. *Frontiers of Oral Biology*.

[117] Atamas S. P., Chapoval S. P., Keegan A. D. (2013). Cytokines in chronic respiratory diseases. *F1000 Biol Rep*.

[118] Konkel J. E., O'Boyle C., Krishnan S. (2019). Distal consequences of oral inflammation. *Frontiers in Immunology*.

[119] Mathew T., Casamassimo P. S., Wilson S., Preisch J., Allen E., Hayes J. R. (1998). Effect of dental treatment on the lung function of children with asthma. *The Journal of the American Dental Association*.

[120] Schmalz G., Arenholt-Bindslev D. (2009). *Biocompatibility of Dental Materials*.

[121] Seng G. F., Gay B. J. (1986). Dangers of sulfites in dental local anesthetic solutions: warning and recommendations. *The Journal of the American Dental Association*.

[122] Housholder G. T., Chan J. T. (1993). Tooth enamel dust as an asthma stimulus: a case report. *Oral Surgery, Oral Medicine, and Oral Pathology*.

[123] Choudat D. (1994). Occupational lung diseases among dental technicians. *Tubercle and Lung Disease*.

[124] Agado B. E., Crawford B., DeLaRosa J. (2012). Effects of periodontal instrumentation on quality of life and illness in patients with chronic obstructive pulmonary disease: a pilot study. *American Dental Hygienists' Association*.

[125] Zhou X., Han J., Liu Z., Song Y., Wang Z., Sun Z. (2014). Effects of periodontal treatment on lung function and exacerbation frequency in patients with chronic obstructive pulmonary disease and chronic periodontitis: a 2-year pilot randomized controlled trial. *Journal of Clinical Periodontology*.

[126] Kucukcoskun M., Baser U., Oztekin G., Kiyan E., Yalcin F. (2013). Initial periodontal treatment for prevention of chronic obstructive pulmonary disease exacerbations. *Journal of Periodontology*.

[127] de Oliveira A. C., Maluta R. P., Stella A. E., Rigobelo E. C., Marin J. M., Ávila F. A. . (2008). Isolation of Pseudomonas aeruginosa strains from dental office environments and units in Barretos, state of São Paulo, Brazil, and analysis of their susceptibility to antimicrobial drugs. *Brazilian Journal of Microbiology*.

[128] Scannapieco F. A., Bush R. B., Paju S. (2003). Associations between periodontal disease and risk for nosocomial bacterial pneumonia and chronic obstructive pulmonary disease. A systematic review. *Annals of Periodontology*.

[129] Brown B., Ma H., Dunbar J., Macewan D. (1963). Foreign bodies in tracheobronchial tree in childhood. *Journal of the Canadian Association of Radiologists*.

[130] Davis C. M. (1966). Inhaled foreign bodies in children: an analysis of 40 cases. *Archives of Disease in Childhood*.

[131] Nadas A. S., Scully R. E. (1965). Case 29-1965. *The New England Journal of Medicine*.

